# Quantum cyber-physical systems

**DOI:** 10.1038/s41598-022-11691-x

**Published:** 2022-05-13

**Authors:** Javier Villalba-Diez, Ana González-Marcos, Joaquín Ordieres-Meré

**Affiliations:** 1 Hochschule Heilbronn, Fakultät Management und Vertrieb, Campus Schwäbisch Hall, 74523 Schwäbisch Hall, Germany; 2 Complex Systems Group, Universidad Politécnica de Madrid, Av. Puerta de Hierro 2, 28040 Madrid, Spain; 3 Department of Mechanical Engineering, Universidad de La Rioja, 26004 Logroño, Spain; 4 Escuela Técnica Superior de Ingenieros Industriales (ETSII), Universidad Politécnica de Madrid, José Gutiérrez Abascal 2, 28006 Madrid, Spain

**Keywords:** Quantum simulation, Qubits

## Abstract

This paper aims to promote a quantum framework that analyzes Industry 4.0 cyber-physical systems more efficiently than traditional simulations used to represent integrated systems. The paper proposes a novel configuration of distributed quantum circuits in multilayered complex networks that enable the evaluation of industrial value creation chains. In particular, two different mechanisms for the integration of information between circuits operating at different layers are proposed, where their behavior is analyzed and compared with the classical conditional probability tables linked to the Bayesian networks. With the proposed method, both linear and nonlinear behaviors become possible while the complexity remains bounded. Applications in the case of Industry 4.0 are discussed when a component’s health is under consideration, where the effect of integration between different quantum cyber-physical digital twin models appears as a relevant implication.

## Introduction

Cyber-physical systems (CPS) are integrations of computational and physical components that can interact with humans through new and different modalities. A key to future technological development is precisely this new and different capacity of interaction together with the new possibilies that these systems pose for expanding the capabilities of the physical world through computation, communication and control^1^. When CPS are understood within the industrial practice fueled by additional technologies such as Internet of Things (IoT), people refer to the Industry 4.0 paradigm^2^. The design of many industrial engineering systems has been performed by separately considering the control system design from the hardware and/or software implementation details. That is, after a first design and verification stage in which the control system is exhaustively simulated, the uncertainty of the generated model and random disturbances are addressed by means of ad hoc adjustment methods, which has been time-consuming and costly in the maintenance of functional and operational systems when several subsystems are integrated^1^.

Leading Industry 4.0 processes to the coordinated achievement of objectives is a probabilistic process in which decision–makers can never be certain that the choice being made is the correct one^3^. As a consequence, value-creating networks can be considered as decision networks or probabilistic directed acyclic graphical models^4,5^ with known conditional probabilities, and such network per process, when considered as an ensemble, is nothing else than a multiple complex system. Multiple complex systems can be found as well in many other fields such as the human nervous system^6^, forests^7^, city transportation systems^8^, social networks^9^ or insect colonies^10^, which present a multilayered hierarchical network structure. The emergence of this type of configuration confers on the system a series of evolutionary fitness advantages^11^ such as the development of distributed swarm intelligence^12^, systemic learning through information aggregation^13^, effective goal achievement and complex problem solving^14^, or greater resilience to changes^15^. Indeed, the strategic design of organizations takes such complexity into account^16^ for cyber-physical systems of Industry 4.0^17^, where they start to be understood as socio-technical complex networked configurations at various levels of complexity^18–21^.

The behavioral simulation of individual systems in Industry 4.0 is frequently addressed by means of digital twins (DTs). Although the DT concept is well established, it is usually crafted in different ways depending on the application and discipline, and they have the vision for representing physical assets, allowing different component models^22^. Most of the efforts in creating DTs are spent in gathering data and training models. However, little efforts have been done to exploit the hierarchical relationship between systems in an integrated way. Therefore, a significant gap regarding DTs is the lack of integration^23^, not only at the same level, losing the capability of promoting their interactions fostering the information value chain, but even vertically, where the upper hierarchical levels are not aware of the status of subsystems or components, relevant for their processing status. The main reason is because of the high complexity involved, which makes it hard to consider all components at all hierarchical levels.

Quantum computation processes information using the laws of quantum mechanics, which endows it with a high computational capacity compared to classical computers^24^. It has opened new ways of solving some problems, e.g., in machine learning^25^, finance^26^, or human interaction^27^. Industry 4.0 problems using machine learning are likely to benefit from quantum models of computation^3^. A quantum simulation may also be used to optimize the configuration of the cyber-physical resources that conform to these systems. It has already been shown how quantum simulations can be used to describe networks of interdependent resources^28–30^.

While a CPS has both physical and software components deeply intertwined in such a way it operates on different spatial and temporal scales, interacting with each other in ways that change with context, we define Quantum Cyber Physical Systems (QCPS) as a CPS in which its mechanism is controlled or monitored by quantum-based algorithms.

**Figure 1:**
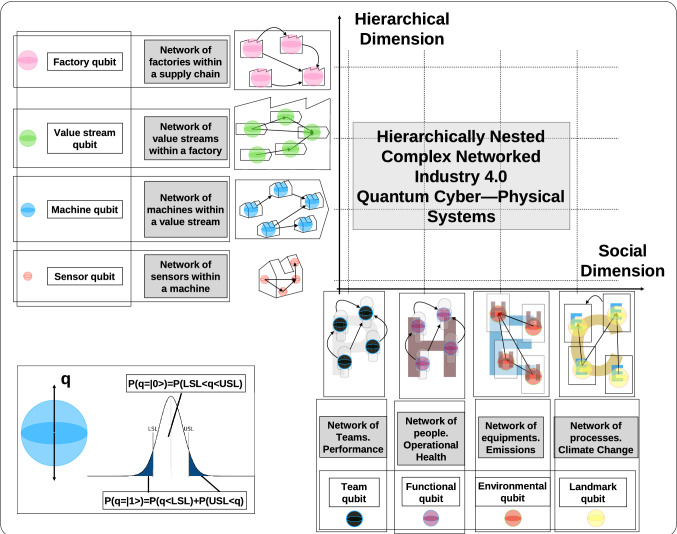
Quantum industry 4.0 cyber-physical systems.

In this article, we combine the system theory as well as its digital translation and the use of quantum decision networks to propose a quantum framework for Industry 4.0 cyber-physical systems, able to deal with the hierarchical interdependent DT. As shown in the graphical abstract of Fig. 1, we propose a two-dimensional socio-technical multilayered network framework in which the resources that make up the cyber-physical system are modeled as a network of qubits or, in their simplest configuration, through qubits. Each of these elements presents a probability of being in the desired process specifications that is represented by the qubit position.

To clarify the proposed architecture, for the exhibited example in the Fig. 1, it is important to understand that the interest was to model and to represent two different behaviors for the elements or subsystems, such as normal (inside the expected range) or abnormal (outside of it). Such a status can be described as a probability at a specific time and it can be represented by a quantum bit (qubit), in a similar way to other approaches that use quantum computing to simulate diffusion through networks^31^.

Therefore, such representation is used on the technical axis to describe the status of a sensor or network of sensors, of a machine or network of machines, of a value stream of resources, or a network of value streams, etc.

On the other axis, this time talking about the nontechnical human or social dimensions of the organization, different interests can be also described in a probabilistic way. In the case of a team (or a network of teams), we could be interested in estimating their performance, or their alignment as normal (inside the expected range through time) or abnormal. However, the focus could be a group of people, where the relevant parameter being described in a probabilistic way could be their operational health, or other dimensions. Indeed, the interest can also be the effect of devices or processes over social agents at a short scale (emissions) or at a larger scale (contribution to climate change).

Each system component is independent and connected to others as defined by the users, facilitating the assembly of a multilayered network structure. This allows users to assess different strategies according to various situations or configurations. In the example used as reference, the interest is to model how abnormalities at specific level impact in higher levels of hierarchy. Furthermore, the paper develops methods to evaluate the effects of resource or system failure at a specific level as well as its propagation and impact. This proposal can be seen as another perspective of the utilization of quantum computing, a similar hierarchical perspective presented by Ref.^32^ in their vision for Quantum Internet.

This paper is organized as follows. Second Section describes quantum multilayered networks. The third section evaluates the implementation of the hierarchical relationship. The fourth section discusses the results. Finally, the fifth section presents our conclusions, limitations, and future research steps.

## Quantum multilayered networks

Reading the proposed framework, each of the two dimensions of the socio-technical phase space can be understood as a specific space, then different layers are considered inside each space. We have separated the aggregation levels as follows:

- At the social level, each layer is defined by a network of agents reporting to each other^33^, where performance, operational health, emissions, and carbon footprint have been identified^22,34,35^ as core elements.
- Similarly, at the technical level, the machine state is defined by a network of sensors^36^, the value stream state is defined by a network of machines^37^, the factory state is defined by a network of value streams^38^, and the supply chain state is defined by a network of factories defining the supra-organizational state^39,40^.

In general, we can state that Industry 4.0 systems can be designed as a network of processes^41^ and the failure of one of these processes leads to a performance loss of the system^42^. We aim to inspect the behavior of the system when some of its elements are not operating within the standard operating procedure tolerances^43^. Industrial processes are designed following strict standard operating procedures that constrain them within certain limits, to ensure the competitiveness and quality of the products^44,45^.

The information describing Industry 4.0 systems is typically managed by a series of key performance indicators (KPIs). Such KPIs are interdependent and describe certain characteristics of the processes that conform to the value creation of certain products^46^. To ensure the necessary conditions of quality, cost, etc., demanded by customers, these KPIs and their respective statistical distributions are constantly monitored by control processes. Our proposal assigns one qubit $q$ to each of the cyber-physical resources of the multilayered socio-technical network. Specifically, the probability that the cyber-physical resource is within the specifications defined in the standard operating procedure will be assigned as the probability of $P(q=|0\rangle )$ and $P(q=|1\rangle )$ otherwise (see Fig. 1). These resources will be linked to others to build a system, depending on the aspect under consideration in such a way that *multilayer network* becomes a natural architecture. Indeed, the state space of a composite physical system is the tensor product of the state spaces of the component physical systems^47^, and this principle can be easily translated to the quantum environment. Therefore, in this work it is proposed a hybrid framework for analyzing classical data coming from different sources through quantum networks.

Bloch’s sphere is commonly used to geometrically represent a qubit^48^. A qubit can be represented as a point on the Bloch sphere with the help of two parameters ($\theta$, $\phi$), as expressed by $|\Psi \rangle =cos\left( \frac{\theta }{2} \right) |0\rangle + e^{i\phi }sin\left( \frac{\theta }{2} \right) |1\rangle$. When several qubits are utilized in a circuit describing a layer of $M$ elements, their aggregated state can be determined utilizing the tensorial product given by $|\Psi \rangle =|\Psi _{1}\rangle \otimes |\Psi _{2}\rangle \otimes ...\otimes |\Psi _{M}\rangle$^47^. This tensorial product maps the entry $x \in \mathbb {C}^n$ in a complex Hilbert space $\mathscr {H}$, which for $n$ qubits is the $\mathbb {C}^{2^{n}}$. For simplicity reasons, we will. For the sake of simplicity, we will henceforth dispense with the notation by mentioning the entry $x$.

A multilayer network $\mathscr {M}$ is given by the quadruplet $\mathscr {M}=(\Gamma _{M},\mathscr {E}_{M},V ,\mathscr {L})$, in which $\Gamma _{M}$ indicates the set of node-layer tuplets related to a set of nodes $V$, and $\mathscr {L}$ representing the set of perspectives built upon a set of elementary layers being relevant to the set of aspects $\mathscr {A}$^49^. Therefore, a multilayer network can have N$_{A}$ number of aspects, being N$_{A}$ the cardinality of $\mathscr {A}$. Based on those aspects a sequence of sets of layers is defined as $\mathscr {L}$ = $\{$ L$_{\alpha }$ $\}$, $\alpha \in (1,2,\dots$,N$_{A}$) , where L$_{\alpha }$ represents the set of layers related to the $\alpha$ aspect. The whole group of layers are built based on the cartesian product of the sets as per aspect, $L_{1} \times L_{2} \times \dots \times L_{N_{A}}$, and then $\Gamma _{M} \subseteq V \times L_{1} \times L_{2} \times \dots \times L_{N_{A}}$. The nodes can be connected pairwise by means of edges, $\mathscr {E}_{M} \subseteq \Gamma _{M} \times \Gamma _{M}$ where according to the definition, connections can happen inside layers or inter-layers. The multilayer network can still be represented as graph $\mathscr {G}_{M} = (\Gamma _{M},\mathscr {E}_{M})$.

For any given time $t$ complex cyber-physical networks have been formally described^21^ as time-dependent *graphs* given by Eq. (1):

1 $$\begin{aligned} \mathscr {G}_{M}(t)=(\Gamma _{M}(t),\mathscr {E}_{M}(t)), \end{aligned}$$

which can be understood as lists of $\Gamma _{M}(t)$ human and cyber-physical nodes and its standard communication $\mathscr {E}_{M}(t)\subset (\Gamma _{M}(t)x\Gamma _{M}(t))$ edges^43^. Continuous improvement-oriented standardization of business communication protocols between network elements (i.e., organizational network edges) is the only way to produce lean structural networks from complex networked organizational design configurations^43^

Within this time interval $\Delta t$, the graph $\mathscr {G}_{M}(t)$ described in Eq. (1) converts into a decision network $\mathscr {G}_{M}(t)'=(\Gamma _{M}(t)',\mathscr {E}_{M}(t)')$ formed by a set of $\Gamma _{M}(t)'$ nodes and $\mathscr {E}_{M}(t)'$ edges, where $\Gamma _{M}(t)'=(\gamma _{M1}(t), \gamma _{M2}(t), \ldots , \gamma _{MN}(t))$ represents the set of all the nodes being part of the network in $\Delta t$, and the edges represent interactions between nodes. Let $\gamma _{Mi}(t)$ be a *parent* node and $\gamma _{Mj}(t)$ a child node. The edge between nodes $\gamma _{Mi}(t)$ and $\gamma _{Mj}(t)$ are determined by the known probabilistic dependence occurrence on node $\gamma _{Mj}(t)$ related to the occurrence on $\gamma _{Mi}(t)$. Subsequently, as described by Ref.^50^, the joint probability of the nodes can be decomposed into the product of the marginal probabilities given by Eq. (2):

2 $$\begin{aligned} P(\Gamma _{M1}(t),\Gamma _{M2}(t), \ldots , \Gamma _{MN}(t))=\prod _{i=1}^{N}P\left( \Gamma _{Mi}(t)| \Gamma _{Mi+1}(t), \ldots , \Gamma _{MN}(t)\right) . \end{aligned}$$

This property shall be used later on for a proper representation of lean complex cyber-physical networks through quantum circuits.

These qubits form decision networks that can be simulated with quantum circuits^3,27,29,30^. In this approach, an amplitude encoding feature map is implemented. For a root node (qubit) with no parents—i.e., it does not depend on any other node, there are two possible states: $|0\rangle$ and $|1\rangle$. The initialization of these qubits is implemented by translating the conditional probabilities depending on their decision network dependencies into *qubit* rotation angles. Therefore the rotation angle $\theta$ required to calculate the probabilities of being in state $|0\rangle$ and $|1\rangle$ can be expressed by Eq. (3):

3 $$\begin{aligned} \theta =2*atan\left[ tan\left( \frac{\theta }{2} \right) \right] =2*atan\sqrt{\frac{sin^2\left( \frac{\theta }{2} \right) }{cos^2\left( \frac{\theta }{2} \right) }}=2*atan\sqrt{\frac{p(|1\rangle )}{p(|0\rangle )}}. \end{aligned}$$

In general, for a child node $\Gamma _{i}$ with m parents there are $2^{m}$ possible states $\prod \Gamma ^{*}_{i}$ and the rotation angle is given by Eq. (4):

4 $$\begin{aligned} \theta _{\Gamma _{i}, \Gamma ^{*}_{i}}=2*atan\sqrt{\frac{p(|1\rangle |\prod \Gamma _{i}= \Gamma ^{*}_{i})}{p(|0\rangle |\prod \Gamma _{i}= \Gamma ^{*}_{i})}}. \end{aligned}$$

This rotation angle can be seen as a special case of the rotation angle proposed by Ref.^51^ for initializing a quantum register to an arbitrary superposed quantum state and used in^52^ for the preparation stage in their estimation on the upper bound of quantum cloning machine. Similarly, the rotation angle given in Eqs. (3) and (4) provides appropriate amplitudes for the basis states $|0\rangle$ and $|1\rangle$.

## Implementation of the hierarchical relationship

According to the already introduced multilayer network configuration, this paper proposes a quantum multilayered network that presents several computational advantages: on the one hand, the state of each qubit can be fully computed as a wave function of the quantum circuit that conforms it at a lower level. This allows for an effective computation of the interactions between different layers and greatly reduces the computational resources needed to elaborate virtual representations of the system as compared with other approaches such as twin factories^53^. On the other hand, it allows for a distributed ledger computation of organizational states and can therefore flexibly and securely evaluate different decision network configurations and aggregate them into greater settings, hence enabling researchers and organizational designers to use advanced quantum simulations to accelerate managerial decision making.

**Figure 2:**
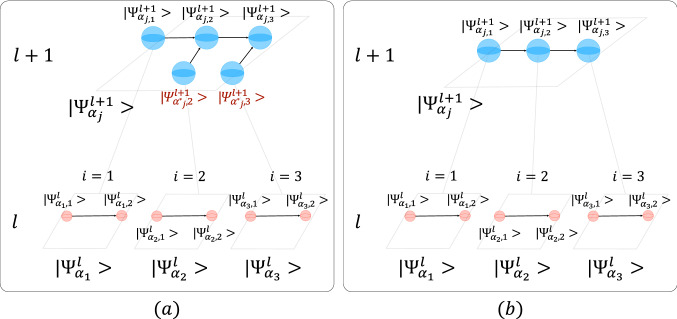
Aggregation of two layers network. Three qubits case. (**a**) First approach (**b**) Second approach. Each node (circle) of the network is represented by a qubit (e.g., sensor qubit in level $l$ and machine qubit in level $l+1$). Interactions between nodes (qubits) are represented by edges in the direction of influence.

Without loss of generality, in the modeling of Industry 4.0 processes, we can always add a qubit at the end of the quantum circuit that measures a characteristic of the circuit that we are interested in measuring (i.e. quality, cost,...) and that condenses the conditional probabilities of the rest of the circuit. As a consequence, the aggregation of the wave function $|\Psi _{\alpha _{j},i}^{l}\rangle$ to the next level $l+1$ in the position $j$ is performed by two nodes: one that describes the initial rotation that represents the $|0\rangle$ probability of the last node of the circuit $P(|\Psi _{\alpha _{j},N_{j}}^{l}\rangle =|0\rangle )$ which absorbs the initial rotation of the level $l$, and a new qubit that contains the conditional probabilities of the node in the level $l+1$. Since the root nodes at level l+1 do not have conditional probabilities, they only present the initial rotation. This is shown in Fig. 2a.

Therefore, the number of additional qubits required to represent the interlayer connections is equal to the number of child nodes in the level $l+1$. For illustration, the quantum circuit at level $l+1$ represented in Fig. 2a uses two additional qubits ($|\Psi _{\alpha ^{*}_{j},2}^{l+1}\rangle$ and $|\Psi _{\alpha ^{*}_{j},3}^{l+1}\rangle$). The initial state of all the qubits in the quantum circuit is $|0\rangle$ (state of no failure). Then, an initial rotation with $\theta$ angles that are conditioned on the states of the last node of the circuits at level $l$ is applied to the root nodes at level $l+1$ and the new qubits that represent the interlayer connections:

- $$P(|\Psi _{\alpha _{j},1}^{l+1}\rangle =|0\rangle ) = P(|\Psi _{\alpha _{1},2}^{l}\rangle =|0\rangle )$$
- $$P(|\Psi _{\alpha ^{*}_{j},2}^{l+1}\rangle =|0\rangle ) = P(|\Psi _{\alpha _{2},2}^{l}\rangle =|0\rangle )$$
- $$P(|\Psi _{\alpha ^{*}_{j},3}^{l+1}\rangle =|0\rangle ) = P(|\Psi _{\alpha _{3},2}^{l}\rangle =|0\rangle )$$.

The calculation of the initial rotation angles is given by Eq. (3).

In the case of child nodes $|\Psi _{\alpha _{j},2}^{l+1}\rangle$ and $|\Psi _{\alpha _{j},3}^{l+1}\rangle$, it is necessary to define a set of probabilities conditioned on the values of the corresponding parent nodes (e.g., $|\Psi _{\alpha ^{*}_{j},2}^{l+1}\rangle$ and $|\Psi _{\alpha _{j},1}^{l+1}\rangle$ for node $|\Psi _{\alpha _{j},2}^{l+1}\rangle$, and $|\Psi _{\alpha ^{*}_{j},3}^{l+1}\rangle$ and $|\Psi _{\alpha _{j},2}^{l+1}\rangle$ for node $|\Psi _{\alpha _{j},3}^{l+1}\rangle$). Thus, $P(|\Psi _{\alpha _{j},i}^{l+1}\rangle =|1\rangle \vert |\Psi _{\alpha ^{*}_{j},i}^{l+1},\Psi _{\alpha _{j},i-1}^{l+1}\rangle =|ab\rangle )$, where $i\in \{2,3\}$ and $|ab\rangle \in \{|11\rangle ,|10\rangle ,|01\rangle ,|00\rangle \}$, represents the probability of failure of node $|\Psi _{\alpha _{j},i}^{l+1}\rangle$ conditioned to the state of failure and/or no-failure of nodes $|\Psi _{\alpha ^{*}_{j},i}^{l+1}\rangle$ and $|\Psi _{\alpha _{j},i-1}^{l+1}\rangle$.

**Figure 3:**
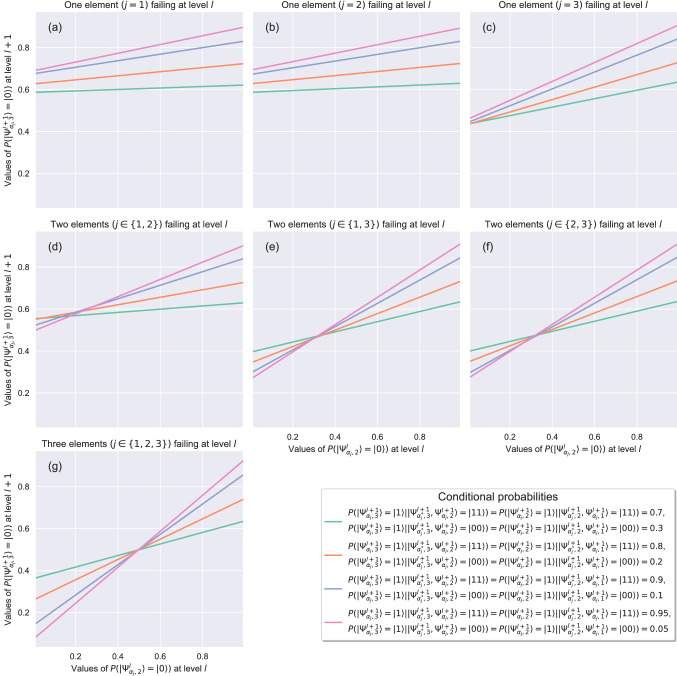
Performance loss of the system at level $l+1$ for different failure behaviors at level $l$, different combinations of $P(|\Psi _{\alpha _{j},i}^{l+1}\rangle =|1\rangle \vert |\Psi _{\alpha ^{*}_{j},i}^{l+1},\Psi _{\alpha _{j},i-1}^{l+1}\rangle =|ab\rangle )$, where $|ab\rangle \in \{|11\rangle ,|00\rangle \}$, and $P(|\Psi _{\alpha _{j},i}^{l+1}\rangle =|1\rangle \vert |\Psi _{\alpha ^{*}_{j},i}^{l+1},\Psi _{\alpha _{j},i-1}^{l+1}\rangle =|10\rangle )=P(|\Psi _{\alpha _{j},i}^{l+1}\rangle =|1\rangle \vert |\Psi _{\alpha ^{*}_{j},i}^{l+1},\Psi _{\alpha _{j},i-1}^{l+1}\rangle =|01\rangle )=0.5$. First approach. Three qubits case.

**Figure 4:**
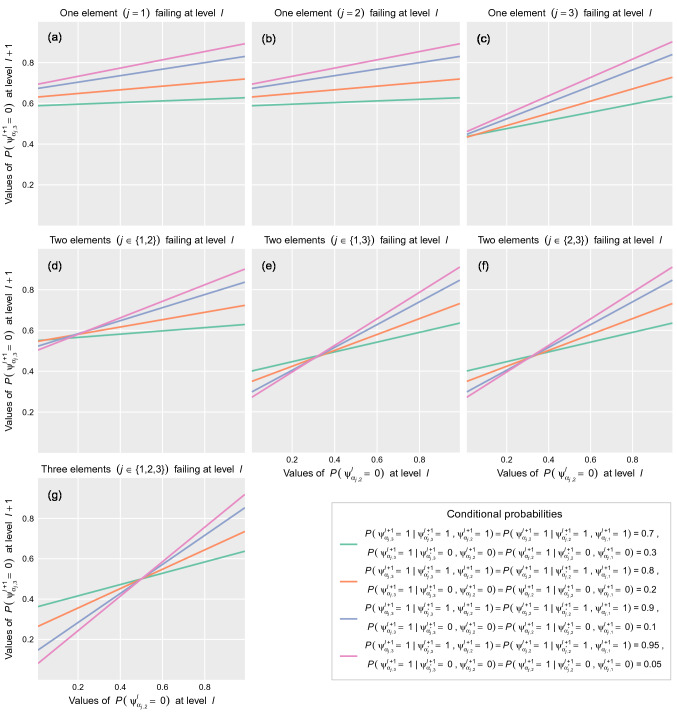
Performance loss of the system at level $l+1$ for different failure behaviors at level $l$, different combinations of $P(\Psi _{\alpha _{j},i}^{l+1}=1\vert (\Psi _{\alpha ^{*}_{j},i}^{l+1},\Psi _{\alpha _{j},i-1}^{l+1})=(a,b))$, where $(a,b)\in \{(1,1),(0,0)\}$, and $P(\Psi _{\alpha _{j},i}^{l+1}=1\vert (\Psi _{\alpha ^{*}_{j},i}^{l+1},\Psi _{\alpha _{j},i-1}^{l+1})=(1,0))=P(\Psi _{\alpha _{j},i}^{l+1}=1\vert (\Psi _{\alpha ^{*}_{j},i}^{l+1},\Psi _{\alpha _{j},i-1}^{l+1})=(0,1))=0.5$. First approach. Equivalent Bayesian network.

The quantum circuit designed with this first approach can be represented by a Bayesian network. Rotation gates in the quantum circuit represent the marginal probabilities associated with root nodes, and controlled rotation gates represent the conditional probability tables associated with child nodes^5^. Thus, the results of the application of this strategy can be compared with the results of the equivalent Bayesian network. As shown in Figs. 3 and 4, the results obtained with both implementations, i.e, the quantum circuit and the classical Bayesian network, are the same. Failure probability propagation from the root nodes—which represent the state of the systems at level $l$—towards the final node—which represents the state of the system at level $l+1$—depends on:

- The set of conditional probabilities that quantifies the effect of the parent nodes on a child. As Fig. 3 illustrates, higher probabilities of failure conditioned on the state of failure of both parent nodes (from $0.7$ to $0.95$ in our example) lead to a greater performance loss of the system.
- The distance between the root node in a state of failure and the final child node. In our example, the root node $|\Psi _{\alpha ^{*}_{j},3}^{l+1}\rangle$ has the greatest impact on the performance loss of the system (see Fig. 3c,e or f).
- The number of root nodes, i.e., systems at level $l$, in a state of failure.

A different proposed approach to connect different layers in the network is to use the state of the last node of the circuit in the level $l$ as initial state of nodes in the level $l+1$ instead of being initialized to $|0\rangle$. In this case, the rotation angle can be expressed by Eq. (5):

5 $$\begin{aligned} \theta =\arccos \left( P\left( |\Psi _{\alpha _{j},N_{j}}^{l}\rangle =|0\rangle \right) \right) . \end{aligned}$$

Thus, $\theta =0$ if the last node of the circuit in the level $l$ has a no-failure state, $(P(|\Psi _{\alpha _{j},N_{j}}^{l}\rangle =|0\rangle )=1)$, and the initial state of the qubits in the level $l+1$ is set to $|0\rangle$, i.e, state of no-failure. On the other hand, as long as the system in the level $l$ is not operating properly $(P(|\Psi _{\alpha _{j},N_{j}}^{l}\rangle =|0\rangle ) \in [0,1))$, the value of $\theta$ increases from $0$ to $\pi /2$, which leads the initial state of the qubits in the level $l+1$ towards a state of failure.

This second approach, which is represented in Fig. 2b, eliminates the use of additional qubits as in the first approach. After the computation of the initial state of nodes in level $l+1$ by applying internal rotation with angles based on Eq. (5), the wave function of the quantum circuit is calculated. To describe the case showed in Fig. 2b, the following probabilities associated with each node need to be defined:

- $P(|\Psi _{\alpha _{j},1}^{l+1}\rangle =|1\rangle )$. Probability of failure of node $|\Psi _{\alpha _{j},1}^{l+1}\rangle$.
- $P(|\Psi _{\alpha _{j},i}^{l+1}\rangle =|1\rangle \vert |\Psi _{\alpha _{j},i-1}^{l+1}\rangle =|a\rangle )$, where $i\in \{2,3\}$ and $|a\rangle \in \{|1\rangle ,|0\rangle \}$. Probability of failure of node $|\Psi _{\alpha _{j},i}^{l+1}\rangle$ conditioned to the state of failure or no-failure of node $|\Psi _{\alpha _{j},i-1}^{l+1}\rangle$.

**Figure 5:**
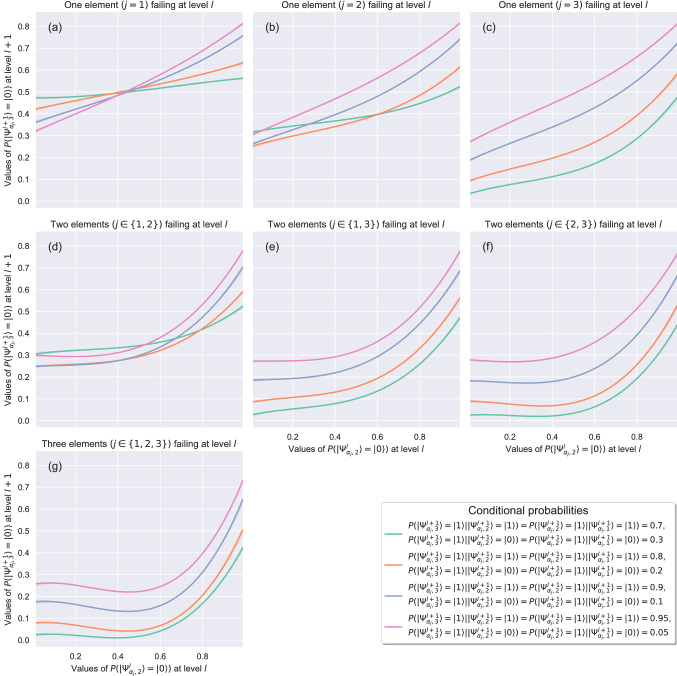
Performance loss of the system at level $l+1$ for different failure behaviors at level $l$ and different combinations of $P(|\Psi _{\alpha _{j},i}^{l+1}\rangle =|1\rangle \vert |\Psi _{\alpha _{j},i-1}^{l+1}\rangle =|a\rangle )$, where $i\in \{2,3\}$ and $|a\rangle \in \{|1\rangle ,|0\rangle \}$. Second approach. Three qubits case.

The results of the application of this second strategy (Fig. 5) show a non-linear behavior of the system when some of its elements are in a state of failure. In this case, the initialization performed by applying internal rotations modifies the initial probability amplitude of quantum states and changes how the information propagates through the quantum circuit. Figure 5 shows how the dominating factors for the performance loss of the system are both the increasing number of nodes (i.e., systems at level $l$) in a state of failure and their decreasing distance to the final node. Furthermore, as the almost parallel lines in Fig. 5c,e,f,g illustrate, the impact of conditional probabilities on the state of the final node decreases with both increasing number of nodes in a state of failure and decreasing distance between failure nodes and the final one, which results in a saturation point where the performance loss of the system becomes flat.

## Discussion

An alternative representation of the quantum state is applied to an entry $x$ $\langle \Psi (x|$ is given by an Hermitian operator $\rho (x)=|\Psi (x)\rangle \langle \Psi (x)|$ called density matrix which contains all the observable information of the quantum state. Quantum circuits map therefore the input into a high-dimensional feature space in which statistical properties of the measurement $M$ are interpreted as output of the quantum circuit. These measurements, which correspond to a Hermitian operator $\mathscr {M}$ acting on vectors in the Hilbert space of the quantum circuit $\mathscr {H}$ and live in a subspace of the data-encoding feature space $\mathscr {F}$ are in general not linear in the Hilbert space $\mathscr {H}$ of the quantum circuit^54^. However, according to the celebrated *representer theorem*^55^, an optimal quantum kernel can be found that allows describing the quantum circuits as linear models in the space of the Hermitian operator $\rho (x)$ with the form $tr\left[ \left( \sum _{m=1}^{M}\alpha _{m}\rho (x^m) \right) \rho (x)\right]$ where $x^m$, $m=1, \ldots ,M$ is the input data and $\alpha _{m}\in \mathbb {R}$. In other words, if we find a linear transformation of our quantum state vector $|\Psi (x)\rangle$, we are guaranteed that the best measurements for our quantum circuit only has $M<<2^{2n}$ degrees of freedom, rather than the $\mathscr {O}(2^{2n})$ degrees of freedom of a quantum circuit with n qubits.

As shown in Fig. 2a, and the related results in Fig. 3, the first quantum model presented in this work does exactly this: by understanding the data-encoding density matrices $\rho (x)$ as feature vectors, it describes a novel quantum kernel that allows the aggregation of hierarchical networks of qubits in such a way that the quantum models behave linearly in the space of the resulting operator when describing the observable information of the quantum state $|\Psi (x)\rangle$. In contrast, the non-linearity of the second model, shown in Fig. 2b, and the related results depicted in Fig. 5, shows dissipating effects in the translation of the failure probabilities from one level $l$ to the next level $l+1$ derived from the internal rotation imposed on the qubits in the hierarchical aggregation^56^. This means that while the circuit has a high-dimensional state space, the quantum model with additional qubits can be operated in a low-dimensional subspace without dissipation, while the model with internal rotations cannot. This representation can be very useful for several applications, but perhaps the most important one is that it allows us to study the temporal evolution of multilayered networked qubit systems given by Eq. (1) from an optimization point of view: minimizing the cost functions represented in the space of quantum circuits would be equivalent to minimizing the same cost functions of the resulting system after the proposed transformation.

## Conclusions

In this paper, a comprehensive framework for articulating different behaviors of components of systems has been proposed. Feasibility of the integrated behavior is obtained through quantum computing circuits describing the connected interaction between elements of those components at every significant level. The proposed framework allows to represent cyber-physical complex networks of components and systems in Industry 4.0 applications, enabling a systematic and quantitative analysis of these systems. This takes into consideration that Industry 4.0 resources can be flexibly activated in different layers inside different processes to create different products. The flexibility of the presented approach comes as it enables the description of different perspectives attending a variety of socio-technical dimensions of the system, preserving its integrated hierarchical description.

The application to the Industry 4.0 case has a particular added value, as it can contribute to efficiently bring vertical and horizontal integration to the digital representation of subsystems, reached through the DT concept, but where every single subsystem on every single dimension requires its particular DT. However, describing the behavior of the whole system requires strong integration capabilities from the quantitative point of view, as more sources of variability need to be considered.

To illustrate the capabilities of the proposed framework, a detailed analysis of the vertical integration between two hierarchical layers of components of a CPS interacting horizontally has been discussed. The adopted criteria of interest are the operational health of the system and its components (non-operating failures), where the failure of components can occur both horizontally and vertically and influence in a different way the system reliability. Without the lack of generalization capability, simple topology configurations were adopted for the multilayered network of components.

This research has also proposed two different effective alternatives for scaling up the aggregated behavior between layers, bringing linear and nonlinear behaviors. The performed simulations associated with this work—quantum simulations have been performed in Python 3.7 and the Bayesian networks have been simulated in R- are available^57^. Explanation of behavior for both alternatives including sensitiveness to failure transmission levels was also undertaken. The results have shown how Industry 4.0 multilayered networks, at any desired scale, can be represented through quantum simulations associated with the conditional probabilities of failure of their cyber-physical elements. They can successfully represent the health of the system, where the computational advantages are relevant, and the possibility of integration of this quantum simulation paradigm in DT Industry 4.0 environments has been demonstrated.

Although Industry 4.0 manufacturing systems are one of the most relevant fields for DTs exploitation, the adoption of DTs can also play a promising role for decision making in alternative fields, such as smart farming or smart cities, among others. Thus, the proposed framework can be easily applied to other contexts, such as organizational lean management, where it can facilitate the representation of communications as well as representing the variability of related processes at different organizational levels.

The adoption of the proposed framework will allow the investigation of alternative descriptions for the components, out of the probability representing its health status, which can include a hybrid representation of interests.

In the future, quantum research on complex hierarchical networks should evolve from the discrete representation of systems as ‘extended qubits’ towards a more ‘continuous’ description through variables representing functions. In mathematical terms, instead of representing as the descriptive basis of the density operator, the complex Hilbert space in which it is located, tends towards more complex Hilbert spaces such as that of bounded L2 functions, or other similar ones. Another pending research topic is the associated optimization problems linked to multilayered networks, where cost functions become defined over the network. Finally, it is worth mentioning that the proposed approach is not a new design of quantum neural networks, such as the many proposals and ideas that can be found in the literature^58,59^. It can be seen as another perspective of quantum complex networks, which have been shown to be more vulnerable to some network attacks than others^60,61^. In this sense, more research is needed to improve information transmission, e.g., quantum spin networks^62^, as well as to allow device independence of security protocols in multisource networks^61^ .
